# Effect of Rocking Movements on Respiration

**DOI:** 10.1371/journal.pone.0150581

**Published:** 2016-03-08

**Authors:** Ximena Omlin, Francesco Crivelli, Lorenz Heinicke, Sebastian Zaunseder, Peter Achermann, Robert Riener

**Affiliations:** 1 Sensory-Motor Systems Lab, ETH Zurich, Zurich, Switzerland; 2 Medical Faculty, University of Zurich, Zurich, Switzerland; 3 Institute of Biomedical Engineering, TU Dresden, Dresden, Germany; 4 Institute of Pharmacology and Toxicology, University of Zurich, Zurich, Switzerland; 5 Zurich Center for Integrative Human Physiology, University of Zurich, Zurich, Switzerland; 6 Neuroscience Center, University and ETH Zurich, Zurich, Switzerland; 7 Zurich Center for Interdisciplinary Sleep Research, University of Zurich, Zurich, Switzerland; Charité - Universitätsmedizin Berlin, GERMANY

## Abstract

For centuries, rocking has been used to promote sleep in babies or toddlers. Recent research suggested that relaxation could play a role in facilitating the transition from waking to sleep during rocking. Breathing techniques are often used to promote relaxation. However, studies investigating head motions and body rotations showed that vestibular stimulation might elicit a vestibulo-respiratory response, leading to an increase in respiration frequency. An increase in respiration frequency would not be considered to promote relaxation in the first place. On the other hand, a coordination of respiration to rhythmic vestibular stimulation has been observed. Therefore, this study aimed to investigate the effect of different movement frequencies and amplitudes on respiration frequency. Furthermore, we tested whether subjects adapt their respiration to movement frequencies below their spontaneous respiration frequency at rest, which could be beneficial for relaxation. Twenty-one healthy subjects (24–42 years, 12 males) were investigated using an actuated bed, moving along a lateral translation. Following movement frequencies were applied: +30%, +15%, -15%, and -30% of subjects’ rest respiration frequency during baseline (no movement). Furthermore, two different movement amplitudes were tested (Amplitudes: 15 cm, 7.5 cm; movement frequency: 0.3 Hz). In addition, five subjects (25–28 years, 2 males) were stimulated with their individual rest respiration frequency. Rocking movements along a lateral translation caused a vestibulo-respiratory adaptation leading to an increase in respiration frequency. The increase was independent of the applied movement frequencies or amplitudes but did not occur when stimulating with subjects’ rest respiration frequency. Furthermore, no synchronization of the respiration frequency to the movement frequency was observed. In particular, subjects did not lower their respiration frequency below their resting frequency. Hence, it was not feasible to influence respiration in a manner that might be considered beneficial for relaxation.

## 1. Introduction

Rocking has been used for centuries to relax babies and toddlers or to promote their sleep [1,2]. Adults also seem susceptible for rocking movements and examples can be found in everyday life situations: commuters easily getting to drowse in a rattling train and many elderly people relax and fall asleep while swinging in a rocking chair. However, the link between rocking movements and relaxation and/or sleep is not well understood until now. Relaxation has been known to have a promoting effect on sleep onset [3]. Bayer et al. [2] suggested that relaxation could also play a role in facilitating the transition from waking to sleep when applying rocking movements. In a recent study, we investigated the influence of different vestibular stimulations (translational and rotational rocking movements) on relaxation [4]. We found that subjects increased their respiration frequency in all conditions with rocking movements compared to baseline, where no movement was applied. Furthermore, subjects’ respiration frequency reached values close to the applied movement frequency of 0.3 Hz. Although, subjects reported to feel relaxed during these movements, an increase in respiration frequency would not be considered to promote relaxation in the first place. Relaxation is characterized by generalized decreased sympathetic nervous system activity, which for instance, leads to and lowering of heart and respiratory rates [5]. Breathing techniques, aiming in lowering the respiratory rate, are widely used to reduce stress and induce relaxation [6]. Furthermore, it was reported that deep and slow breathing reduces blood pressure [7,8] and heart rate [9], and influences autonomic and pain processing [10]. However, as only one movement frequency and one amplitude (0.3 Hz, 15 cm) were applied in the above mentioned study [4], it is not clear what influence other frequencies or amplitudes would have on respiration.

Stimulation of the vestibular system, by electrical stimulation of the vestibular nerve or as a result of postural changes, can elicit respiratory adaptations [11]. The activation of the vestibular system causes reflex modulation of inspiratory and expiratory muscle activity, adjusting respiration and airway patency during head and body movements [11,12]. Essential for these reflexes are the medial and inferior vestibular nuclei [11,13]. Previous studies reported an increase in respiration frequency when applying vestibular stimulation eliciting a vestibule-respiratory responds [14–17]. Jáuregui-Renaud et al. [17] found that rotation in the yaw and pitch planes lead to an increase in respiration frequency in healthy subjects, but not in vestibular deficient patients. In line with this findings, Monahan et al. [14], showed that the stimulation of the semicircular canals, but not the otolith organs or neck muscle afferents, increased ventilation frequency in humans. However, it must be noted that the findings of the aforementioned studies correspond to rotational movements with amplitudes up to 155 degrees. Such large amplitudes would never be reached when applying rocking movements to promote relaxation.

Furthermore, it was proposed that the vestibular system might be partly responsible for the entrainment of respiratory muscles with the underlying pattern of body movements, by coupling the respiratory rhythm with the ongoing movement [15]. Such an entrainment has been observed in various protocols of exercise and active movements [18–22]. Rocking movements have also been used to entrain respiration in premature infants [23]. Sammon et al. [23] used rocking movements to provide a phasic input to the respiratory pattern generator and entrain with it the rocking rhythm. Furthermore, Rassler and Raabe [24] suggested that rhythmic vestibular stimulation can induce coordination with breathing to the same extent as found for active movements.

Thus, the aim of this study was to identify the relationship between the rocking and respiration by investigate the effect of different rocking frequencies and amplitudes on respiration. Knowledge about this relationship could be beneficial for the selection of optimal stimulation parameters in order to promote relaxation using rocking movements. We were interested to see whether subjects would entrain their respiration to rocking frequencies below their spontaneous respiration frequency by breathing slower and deeper. Furthermore, it was of interest to identify whether there are rocking frequencies above subjects’ spontaneous respiration frequency that do not result in a frequency increase and could be used in future relaxation studies. In addition, heart rate and heart rate variability (HRV) were assessed, as due to interactions between vestibular and autonomic pathways, also cardiovascular functions could be influenced by vestibular stimulation [25,26].

## 2. Material and Methods

### 2.1 Subjects

Twenty-one healthy subjects (9 female, 12 male, age: 24–42 years) participated in this study. Subjects were all non-smokers with no medical problems related to the vestibular or respiratory system. Furthermore, subjects had to be well rested (no acute sleep deprivation) and did not suffer from sleep disturbances (assessed by questionnaire). In addition, subjects were not taking any medication for at least 24 hours and caffeinated beverages or food for at least four hours prior to the measurement. All subjects signed an informed consent and the study was approved by the Institutional Review Board of the Swiss Federal Institute of Technology in Zurich (ETH Zurich) (EK 2012-N-39). The study was performed in accordance with the standards for research involving human subjects defined by the Declaration of Helsinki [27].

### 2.2 Device

**Fig 1:**
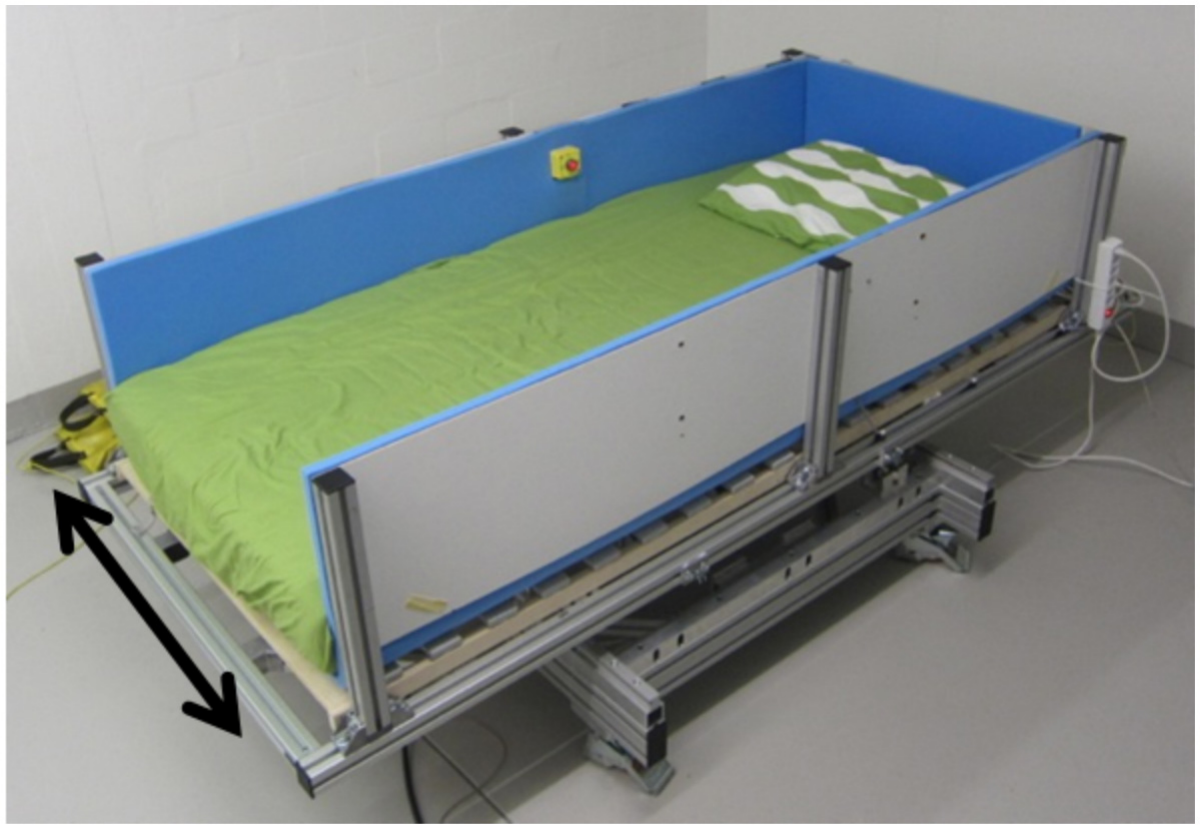
Actuated bed. Actuated bed used to apply the lateral rocking movement. The lateral movement axis is indicated by the arrows.

The measurements were conducted using an actuated bed [28], enabling movements along a lateral axis (Fig 1). The actuated bed was composed of a conventional bed, in form of a mattress and a slatted frame, mounted on a moving platform. While the vestibular stimulation was provided, subjects where lying in a supine position with eyes closed, in a darkened room. The subjects’ head was supported by a foam pillow.

### 2.3 Protocol

**Fig 2:**
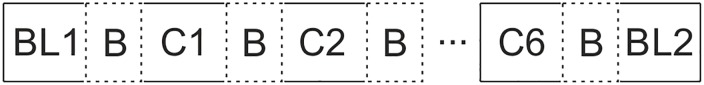
Measurement protocol. Measurement protocol as applied in the study. The conditions were presented in randomized order separated by short breaks. (BL_i_ = Baseline_i_; B = Break; C_i_ = Condition_i_)

Movements along a lateral axis were chosen to avoid rotational movements. Rotational movements were found to activate the vestibulo-respiratory reflex by stimulating the semicircular canals leading to an increase in respiration frequency. However, translation movements stimulating the otolithic organ appear to not influence respiration [14,16,17]. Therefore, only a lateral (translational) movements were applied to reduce the effect of the vestibule-respiratory reflex and to allow a potential entrainment to the movement frequency. Throughout the measurements, four different movement frequencies and two different movement amplitudes were tested in randomized order. In addition, the measurements included two baseline recordings (at the beginning and end), i.e. no movement was applied. The baseline at the end of the measurement was included to test whether the respiration frequency returned to baseline values after the movement had stopped. The two baselines and each movement condition lasted five minutes. Between conditions a short break (3 minutes) without movement was scheduled (Fig 2).

#### Frequencies

The measurement consisted of two conditions with a movement frequency below the subject’s rest respiration frequency and two conditions with a movement frequency above the subject’s rest respiration frequency. Movement frequencies of -30%, -15%, +15% and +30% of subject’s rest respiration frequency were applied. Baseline measurement at the beginning of the session was used to determine each subject’s rest respiration frequency. All movements were performed with an amplitude of 15 cm.

#### Amplitudes

Two different amplitudes, 7.5 cm and 15 cm, were applied with a movement frequency of 0.3 Hz.

### 2.4 Physiological Recordings & Data Analysis

Physiological signals were recorded with the biosignal amplifier g.USBamp (g.tec medical bioengineering, Graz, Austria). The signals were band-pass filtered (0.01–100 Hz) and a 50-Hz notch filter was applied. The signals were sampled with 600 Hz.

Respiration was recorded with a thermistor flow sensor (Thermistor Flow Sensor, S.L.P. Inc., St. Charles, Illinois, USA) placed beneath the nose and a respiration belt (EPM Sytems, Midlothiana, USA) placed around the abdomen. For analysis, the respiration signal was first filtered with a fourth-order Butterworth low-pass filter with a cut-off frequency of 5 Hz. Peaks in the signal were detected with a simple algorithm based on the signal's first and second derivatives. The periods of respiration were determined as the times between two consecutive peaks, mean respiration frequency was calculated as the mean reciprocal value of the respiratory period [29].

ECG was recorded with one electrode placed 2 cm below the right clavicula between the first and second ribs, the second one placed at the fifth intercostal space on the midaxillary line on the left side of the body, and a ground electrode on the right acromion. Mean heart rate was calculated using the NN intervals (intervals between normal beats). Heart rate variability (HRV) parameters were calculated according to the Task Force of the European Society of Cardiology and the North American Society of Pacing and Electrophysiology [30]. To derive the spectral parameters the filtered beat-to-beat intervals were resampled to 4 Hz using the method proposed by Berger et al. [31]. The spectral representation was obtained based on an autoregressive model of 15^th^ order. Besides the total power (defined as the power in the frequency range < = 0.4 Hz), derived powers in the very low frequency range (VLF: < = 0.04 Hz) low frequency range (LF: 0.04–0.15 Hz) and high frequency range (HF: 0.15–0.4 Hz) were calculated using the Biosig Toolbox for Matlab [32].

### 2.5 Statistical Analysis

Physiological variables were statistically evaluated with a univariate general linear model followed by post hoc least significant difference tests (LSD-tests). The conditions with movement were compared among each other and to baseline measurements. Furthermore, baselines (at the beginning and end of the measurement) were compared. Respiration and ECG features were defined in the model as dependent variable, condition as fixed effect and the factor subject as random effect. The significance level was set at p < 0.05. The statistical analysis was performed with SPSS (SPSS Inc., Chicago, Illinois, USA).

### 2.6 Supplementary Study

In addition to the main study five healthy subjects (3 females, 2 males, age: 25–28) were measured using their individual respiration frequency at rest as stimulation frequency. Rocking with subjects’ respiration frequency at rest was not performed in the main study and was therefore investigated in this supplementary study to facilitate interpretation. In order to avoid bias or intentional respiratory adaptations to the movement frequency, we decided to measure naïve subjects (main study participants were informed about the hypothesis of the study after successful completion of the measurements). The study consisted of a baseline measurement and one stimulation condition (5 minutes each). Respiration frequency was measured and analyzed using the same methods as in the main study.

## 3. Results

Respiration frequency in the main study did not differ significantly between the two baselines (Baseline at the beginning: 0.228 ± 0.062 Hz (mean ± SD); baseline at the end: 0.222 ± 0.057 Hz; p = 0.723). Hence, in the following, conditions with rocking movements were compared to the baseline at the beginning of the measurements.

### 3.1 Respiration

#### Effect of movement frequencies

**Fig 3:**
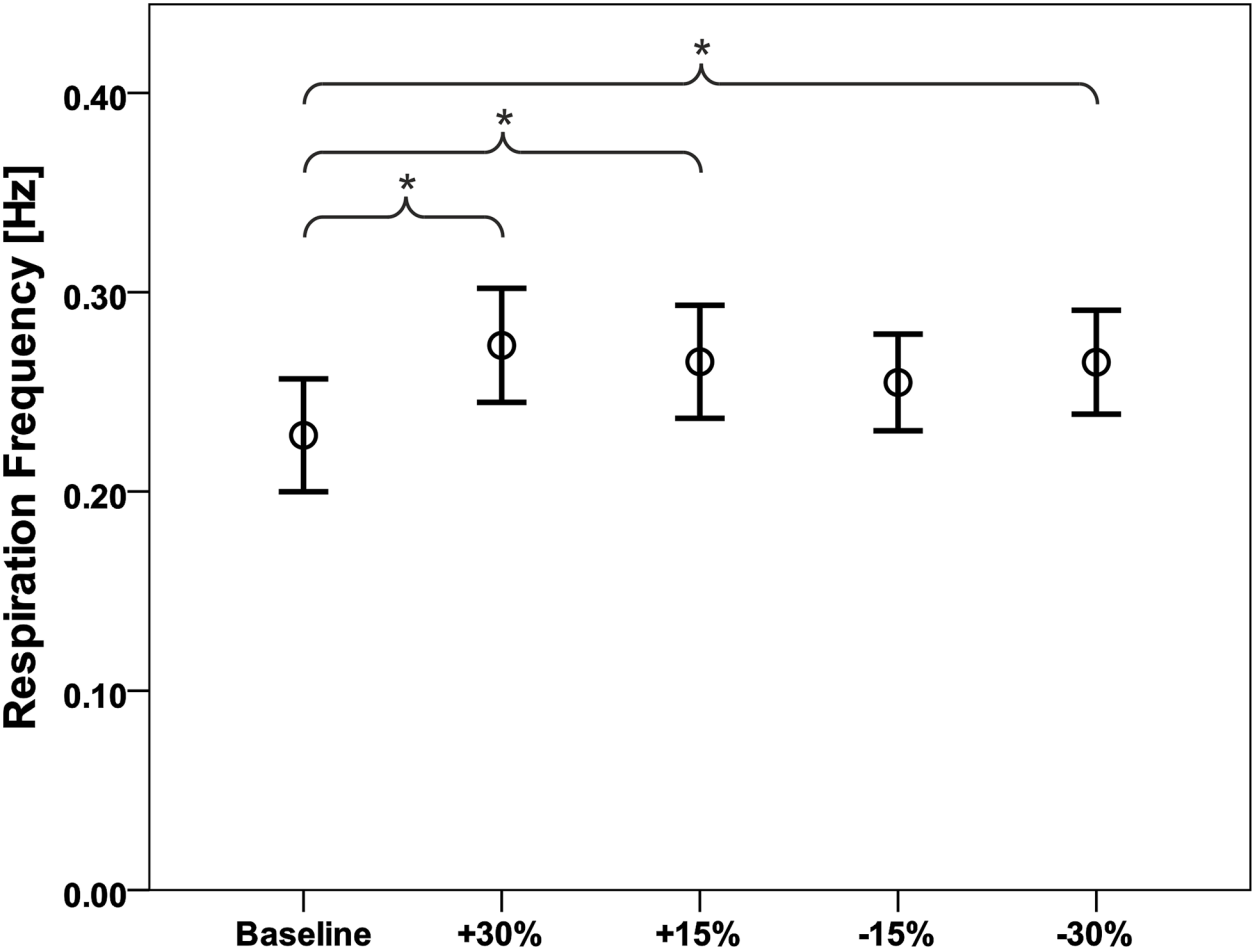
Respiration frequency for the different movement frequencies. Mean respiration frequency [Hz] and standard deviation of the baseline and the four movement frequencies applied. (* p < 0.05)

The applied movement frequencies ranged from 0.1–0.4 Hz. Respiration frequency was increased for all tested movement frequencies compared to baseline (0.228 ± 0.062 Hz; mean ± SD; Fig 3). The increase was significant for the conditions: +30% (0.273 ± 0.063 Hz, p = 0.014), +15% (0.265 ± 0.062 Hz, p = 0.043) and -30% (0.263 ± 0.056 Hz, p = 0.047). The percentage increase from baseline was 28.75% (+30% condition), 23.07% (+15% condition), 23.07% (-15% condition) and 25.71% (-30% condition). The resulting respiration frequencies did not differ between conditions.

#### Effect of movement amplitudes

**Fig 4:**
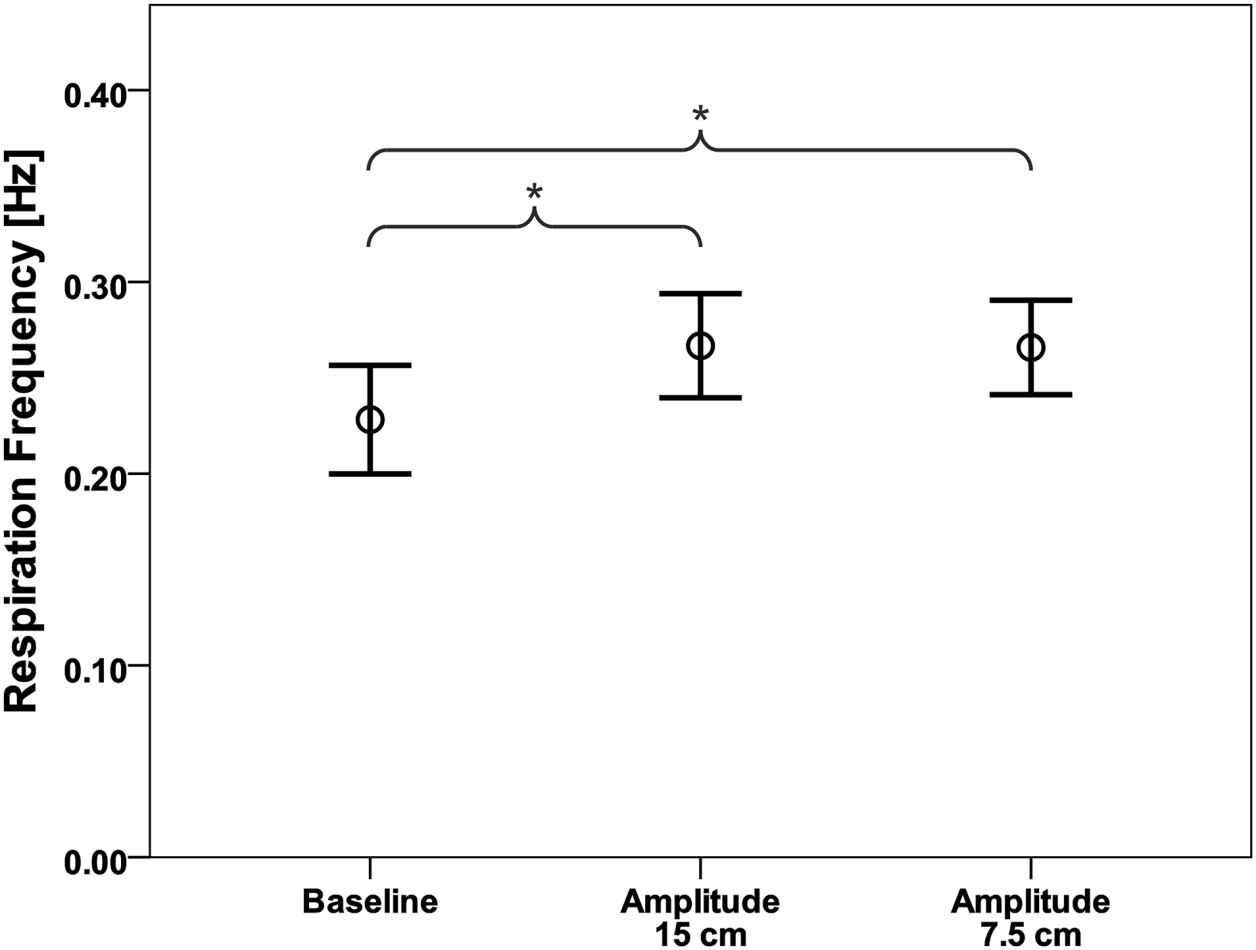
Respiration frequency for the different movement amplitudes. Mean respiration frequency [Hz] and standard deviation of the baseline and the two movement amplitudes applied. (*p < 0.05)

A significant increase in respiration frequency compared to baseline (0.228 ± 0.062 Hz) was found for movement amplitudes of 15 cm (0.267 ± 0.060 Hz, p = 0.039) and 7.5 cm (0.266 ± 0.054 Hz, p = 0.035; Fig 4). The percentage increase from baseline for the 15 cm amplitude was 23.84% and for the 7.5 cm amplitude 27.17%. However, no differences between resulting respiration frequencies of the two stimulation amplitudes were observed.

#### Rocking with subjects respiration frequency at rest

Respiration frequency did not differ between baseline (0.266 ± 0.015 Hz) and the movement condition (0.258 ± 0.010 Hz), during which the movement frequency corresponded to the respiration frequency at rest (Table 1).

**Table 1:** Results of supplementary study. Respiration frequency during baseline and movement condition of all subjects.

Subject	Baseline (Hz)	Movement Condition (Hz)	Percentage Difference [%]
1	0.253	0.258	1.78
2	0.242	0.214	11.50
3	0.237	0.218	8.02
4	0.298	0.296	0.61
5	0.299	0.304	1.62
**Mean**	**0.266**	**0.258**	**4.70**

### 3.2 Heart Rate and Heart Rate Variability

Heart rate significantly decreased in the movement conditions compared to baseline (Table 2). The LF component of HRV was lower than baseline for the movement conditions with a frequency of +30%, -15%, -30% and an amplitude of 15 cm. The remaining HRV variables did not differ from baseline.

**Table 2:** Mean values and standard deviation for heart rate and heart rate variability of all conditions.

	Baseline	Amplitude 15 cm	Amplitude 7.5 cm	+30%	+15%	-15%	-30%
**Heart Rate (bpm)**	67.1 (9.5)	64.6* (8.1)	63.9*(7.0)	64.1* (8.3)	64.6* (9.0)	64.2*(8.2)	64.4* (8.4)
**Total Power (ms**^**2**^**)**	1148.3 (854.8)	1118.4 (758.5)	1277.2 (856.7)	1130.9 (827.8)	1333.1 (1088.1)	1065.4 (633.1)	1199.7 (706.0)
**VLF (ms**^**2**^**)**	275.0 (189.9)	442.3 (447.9)	540.3 (446.5)	419.0 (347.8)	509.9 (635.2)	431.6 (423.6)	478.6 (313.1)
**LF (ms**^**2**^**)**	525.0 (505.2)	368.3* (254.3)	420.3 (279.5)	381.4* (279.5)	497.1 (207.9)	340.7* (207.9)	390.6* (235.7)
**HF (ms**^**2**^**)**	327.7 (315.0)	289.1 (242.9)	294.4 (221.2)	311.6 (272.1)	302.2 (259.6)	276.8 (222.0)	310.7 (263.5)
**LF/HF**	2.1 (1.6)	1.9 (1.8)	2.1 (2.5)	1.8 (1.9)	2.2 (2.2)	1.8 (1.7)	2.0 (2.5)

Very low frequency range (VLF: < = 0.04 Hz); low frequency range (LF: 0.04–0.15 Hz); high frequency range (HF: 0.15–0.4 Hz);

* = p<0.05 (LSD-test), significantly different from baseline

## 4. Discussion

The present study examined the effect of different rocking frequencies and amplitudes on respiration. Our data demonstrate that also movements along a lateral translation can activate a vestibulo-respiratory adaptation causing an increase in respiration frequency in subjects lying in a supine position. This increase might be related to the activation of the vestibular system causing a reflex modulation of the respiratory functions [11,12]. An increase in respiration frequency due to vestibular stimulation has previously been reported [14–17]. Changes in respiration were found when applying head rotations with amplitudes of at least 60°, however, not with translational movements. In the present study a lateral translation of the whole body (in a supine position), without any rotational movement was used as stimulation. Nevertheless, small head movements cannot be ruled out completely as subjects’ head was positioned on a foam pillow to support the head, though it was not fixed. However, if head motions would have occurred, the amplitude of motion would have been in a small range and not comparable with the head rotations of the aforementioned studies.

Any movement frequencies, other than the respiration frequency at rest resulted in an increase in respiration. However, the increase was independent of the movement frequencies (+30%, +15%, -15%, -30% of rest) or amplitudes. Therefore, it appears that any stimulation differing from respiration at rest increases the respiration frequency independent of the applied frequency or amplitude. Since the movement frequencies were adapted to each subject individually, actual stimulation frequencies cannot be compared between subjects. However, movement frequencies ranging from -30% (of the resting respiration frequency) up to +30% did alter respiration in a similar way. Hence, a synchronization of the respiration frequency to the movement frequency did not occur. Therefore, it was not feasible to influence respiration in a manner that might be considered beneficial for relaxation. In particular, since it was not possible to lower respiration frequency below the respiration frequency at rest with a slower stimulus frequency.

A potential explanation for this finding might lie in the fact that entrainment of respiration frequency in adults was mainly found for voluntary movements [20,33–36]. In contrast, our movements were passively induced. Nevertheless, Rassler and Raabe [24] observed a coordination of respiration to the passive turning in a swivel-chair. However, this passive turning involved a rotation of the head, which was not present in our lateral translation. Furthermore, Sammon and Darnall [23] did not find entrainment of respiration frequency for all rocking frequencies. The authors concluded that although respiration did not entrain to the rocking, it does not mean that the vestibular input didn’t stimulate respiration. Hence, the lateral movements used in our study seem to stimulate the respiratory system, though it did not lead to an entrainment. Remarkably, respiration was only affected when the stimulation did not match subjects’ respiration frequency at rest. Only deviating frequencies appear to represent strong enough stimuli to trigger adaption of the respiratory system. However, to explain these findings in detail, further investigations with a larger study sample are needed. Nevertheless, it was not possible to identify stimulation parameters that lowered respiration frequency and thus could influence relaxation positively during rocking. Our findings indicate that an increase in respiration frequency has to be expected when applying rocking movements (deviating from subjects resting respiration frequency) to promote relaxation and potentially facilitate sleep onset. In a next step, it needs to be investigated whether this increase in respiration frequency is a transient phenomenon. We might not have measured long enough to see whether the respiration frequency returned to resting levels. However, as both baseline measurements (at the beginning and end of the measurement) did not differ significantly it can be assumed that respiration frequency returns to baseline values once vestibular stimulation is stopped. Furthermore, it has to be considered that no electroencephalogram (EEG) data were measured. Therefore, it cannot be ruled out completely that subjects fell asleep during the different conditions, even though we don’t expect it to be very likely. Subjects were well rested (see inclusion criteria section 2.1. Subjects) and measurement were conducted throughout the afternoon. In addition, the short condition duration of five minutes was chosen to reduce the likeliness for subjects to fall asleep.

Although stimulation led to an increase in respiration frequency, heart rate decreased in the conditions with movement compared to baseline. However, previous studies stimulating the vestibular system via postural changes of the head or body reported contradictory findings with respect to cardiovascular functions. Thurrell et al. [15] found no changes in heart rate for whole body oscillation in the yaw plane after standing, whereas changes in heart rate were reported for head down rotation in both young and older adults [14,16]. Furthermore, alterations in respiration but no changes in cardiovascular parameters were observed for rotations in the yaw and pitch plan of the head or dynamic upright roll in several studies [14,16,17]. However, the types of stimulation as well as the stimulation parameters differed considerably between the above mentioned studies and it is not yet clear if potential changes in heart rate are related to cardio-respiratory coupling. In our study, the decrease in heart rate might be related to the fact that subjects reported to feel relaxed during the rocking conditions. The changes found in the LF component of HRV would support such a hypothesis as the LF component represents a marker of sympathetic modulation [37]. Therefore, the observed decrease in LF power could indicate a reduction in the sympathetic tone and might not be related to the changes in respiration. However, a clear conclusion, concerning the effect of vestibular stimulation on cardiovascular functions, is difficult to draw and needs further investigation. In particular, procedures beyond the standard HRV analysis should be invoked in order to reveal more complex mechanisms of cardio-respiratory coupling.

## 5. Conclusion

Rocking movements along a lateral translation caused a vestibulo-respiratory adaptation leading to an increase in respiration frequency in subjects lying in a supine position. The increase was independent of the applied movement frequencies or amplitudes but did not occur when stimulating with subjects’ rest respiration frequency. No synchronization to the movement frequencies was observed, hence, it was not feasible to influence respiration in a manner that might be considered beneficial for relaxation. These results suggest that stimulation deviating from subjects’ respiration at rest might lead to an increase in respiration frequency independent off the applied stimulation frequency or amplitude.
